# EBV based cancer prevention and therapy in nasopharyngeal carcinoma

**DOI:** 10.1038/s41698-017-0018-x

**Published:** 2017-05-15

**Authors:** Ya Cao

**Affiliations:** 1 Key Laboratory of Carcinogenesis and Invasion, Ministry of Education, Xiangya Hospital, Central South University, Changsha, 410008 China; 2 Cancer Research Institute, Xiangya School of Medicine, Central South University, Changsha, 410078 China; 3 Key Laboratory of Carcinogenesis, Chinese Ministry of Health, Changsha, 410078 China; 4 Research Center for Technologies of Nucleic Acid-Based Diagnostics and Therapeutics Hunan Province, Changsha, 410078 China

## Abstract

Epstein–Barr virus is an important cancer causing virus. Nasopharyngeal carcinoma is an infection-related cancer strongly driven by Epstein–Barr virus. In this cancer model, we identified the major host targets of latent membrane protein 1 which is a driving oncogene encoded by Epstein–Barr virus in latency infection. latent membrane protein 1 activates several oncogenic signaling axes causing multiple malignant phenotypes and therapeutic resistance. Also, Epstein–Barr virus up-regulates DNA methyltransferase 1 and mediates onco-epigenetic effects in the carcinogenesis. The collaborating pathways activated by latent membrane protein 1 constructs an oncogenic signaling network, which makes latent membrane protein 1 an important potential target for effective treatment or preventive intervention. In Epstein–Barr virus lytic phase, the plasma level of Epstein–Barr virus DNA is considered as a distinguishing marker for nasopharyngeal carcinoma in subjects from healthy high-risk populations and is also a novel prognostic marker in Epstein–Barr virus-positive nasopharyngeal carcinoma. Now the early detection and screening of the lytic proteins and Epstein–Barr virus DNA have been applied to clinical and high-risk population. The knowledge generated regarding Epstein–Barr virus can be used in Epstein–Barr virus based precision cancer prevention and therapy in the near future.

## Epstein–Barr virus (EBV) associated cancers are important global health concerns

The Global burden of cancers in 2008 reached 12.7 million new cancers worldwide. Approximately 1.98 million were attributable to infections, including hepatitis B virus, hepatitis C virus, human papillomavirus (HPV), EBV, and H pylori.^1^ EBV is a human cancer-associated virus that infects >90% of the global population.^2^ EBV was first discovered in cultured tumor cells derived from a Burkitt lymphoma biopsy acquired from an African patient in 1964.^3^

Now EBV is a well-recognized carcinogen that has been implicated in the etiology of several malignancies of both epithelial and lymphoid origins, including gastric cancer, nasopharyngeal carcinoma (NPC), non-Hodgkin’s lymphoma, and Hodgkin’s lymphoma.^4^ EBV-attributable malignancies have increased by 14.6% over a period of 20 years and almost 50% of EBV-attributed malignancies occurred in East Asia.^4^ Now EBV-associated cancers account for approximately 1.5% of all cancers worldwide and represent 1.8% of all cancer deaths.^5–7^ The U.S. National Institutes of Health recently called for a new initiative to reduce global cancer incidence, with EBV among the top candidates for advanced studies.^6^

## EBV-latent membrane protein 1 (LMP1) is a driving oncogene in NPC

As a subtype of head and neck carcinoma, NPC is etiologically related to the EBV infection. EBV can establish latent or lytic phase in the host cells. The both phases are involved in carcinogenesis. And EBV infection in NPC cells is mainly latent. In the latent phase, EBV encodes an onco-protein latent membrane protein 1 (LMP1).^8, 9^ LMP1 expresses in almost all primary NPC tissues.^10^ LMP1 is 386-animo-acid protein. And its C-terminal active region (CTAR) can mediate multiple oncogenic signaling, which contribute to its oncogenic effects.^11^ In NPC, several groups contributed their important discoveries in EBV and LMP1 pathogenesis. They found that LMP1 increases the localization of phosphatidylinositol 3-kinase (PI3K) and Akt to lipid rafts which reorganizes membrane and cytoskeleton microdomains to modulate signal transduction.^12^ Also, LMP1 increases the expression of some cancer progenitor cells (CPC) markers and induced a CPC-like phenotype in epithelial cells.^13^

We also identified the major host targets of LMP1 and its associated malignant phenotypes including cell cycle (Op18, p53, EGFR, STAT3, Cyclin D1), invasion and metastasis (C-X-C motif chemokine receptor 4), metabolic reprogramming (hexokinase 2), angiogenesis (vascular endothelial growth factor), apoptosis (survivin) and epithelial-mesenchymal transition (EMT).^14–24^ Also, we found LMP1 promotes oncogenic phenotypes by multiple post-translational modification, such as phosphorylation, ubiquitination, sulfation, methylation, and sumoylation.^14, 17, 20, 23, 25, 26^ Interestingly, LMP1 CTAR3-Ubc9 interaction modulates sumoylation processes, which regulates signal transduction pathways that affect phenotypic changes associated with oncogenesis and cellular migration.^26^

The major signal pathways activated by LMP1 in NPC is NF-κB, PI3K/AKT, MAPK, and ERK.^8^ Another team also found that LMP1 oncoprotein modulates cell adhesion via the regulation of activin A/TGFβ and β1 integrin signaling.^27^ More importantly, LMP1 activates several oncogenic signaling axes LMP1/JAK3/EGFR/STAT3/VEGF, LMP1/JNKs/c-Jun/HIF-1/VEGF, LMP1/NF-κB/ATM, LMP1/ERK/OP18 which mediate therapeutic resistance.^14, 22, 23, 28, 29^ Notably, one of the phenotype caused by LMP1 is glycolysis. LMP-1 induces the expression of HK2.^29^ And knockdown of HK2 in the LMP1 expressing NPC cells induces cell death, indicating that the induction of glycolysis is necessary for cell survival. The LMP1/PI3K/Akt/GSK3β/c-Myc/HK2 axis is involved in this regulation. Recently, we discovered that a new mechanism of LMP1-induced radioresistance occurring through the modulation of LMP1/DNAPK/AMPK DDR signaling axis and provided a mechanistic rationale supporting the use of AMP-activated protein kinase (AMPK) activators for facilitating NPC radiotherapy.^23^ Together, LMP1 promotes tumorigenesis and therapeutic resistance by inappropriate regulation of key transcription factors and kinases.

And the collaborating pathways activated by LMP1 constructs an oncogenic signaling network, which makes LMP1 an important potential target for effective treatment or preventive intervention.

Now evaluation of LMP1 as a therapeutic target is identified.^30^ As LMP1 is an attractive and promising target for EBV-positive NPC, we successfully developed a DNAzyme (DZ1) that was engineered to specifically target the LMP1 mRNA.^31^ DZ1 treatment can reverse malignant phenotypes caused by LMP1 and increase the radiosensitivity of NPC, indicating the potential for DZ1 therapeutic approaches for the treatment of EBV-related cancers.^23, 31–33^ Recently, an adenovirus-based adoptive immunotherapy has been developed that encodes Epstein–Barr nuclear antigen 1 (EBNA1) fused to multiple CD8 T-cell epitopes from LMP1 and LMP2.^34^ Also, LMP1-specific autologous CTLs were further proven in recurrent NPC patient. These early trials showed promising results of the use of EBV-CTL therapy in NPC patients.^35^

## EBV mediated oncoepigenic effect in NPC

The malignant phenotypes drived by EBV are a combination of genetic and epigenetic changes. The CpG island methylator phenotype is a general phenomenon in cancers and involves different genes depending on the tumor types.^36, 37^ EBV infection is an epigenetic driver. EBV mediated onco-epigenetic effects in NPC can cause multiple tumor suppressing gene silence.^38^ In NPC, more than 60% tumor suppressor genes (TSGs) are silenced by methylation, including death associated protein kinase 1, phosphatase and tensin homolog, E-cadherin, PCDH, IRF8, and p16.^37, 39–42^ These promoter hypermethylation and epigenetic silencing of cellular genes through the activation of DNA methyltransferase 1 (DNMT1) and the polycomb group protein, B lymphoma Mo-MLV insertion region 1 homolog.^43, 44^ In addition to the up-regulation of DNMT1 activity by LMP1, LMP1 also can promote DNMT1 translocation to the mitochondria, which can increase mtDNMT1 expression and inhibit mitochondrial respiration (unpublished data). Up-regulating DNMT1 and driving malignant phenotypes by epigenetic mechanism play a crucial role in NPC, especially in the early stage of this cancer.

The recent development of agents or food components that reverse methylation-induced inactivation of gene expression is a new promising approach for cancer prevention.^45–49^ Restore methylated and silenced TSGs could be expected to recover phenotypes of tumor cells. A clinical trial of azacitidine was carried out in patients with NPC and EBV positive AIDS-associated Burkkit’s lymphoma.^35^ We found that a natural compound grifolin from *albatrellus confluens*, can decrease the transcription activity and expression of DNMT1, and restore mitochondrial respiration in LMP1-positive NPC cells (unpublished data). Targeting both EBV and DNMT1, which are predominantly expressed in the NPC early stage, may be a good strategy in the EBV-positive cancer’s prevention and therapy.

## EBV reactivation and oncogenesis in NPC

Researchers paid their major attention to the transformation role in EBV latency infection for a long time. Evidence of a contribution of the lytic cycle to EBV-induced oncogenesis has emerged only in recent years.^11^ EBV lytic phase is associated with NPC, GC and multiple lymphomas and confers therapeutic resistance to tumor cells.^50, 51^ The expression of lytic EBV genes was observed in the upper layers of differentiated epithelium but not in the undifferentiated basal layers.^52^ In NPC patients, high levels of lytic proteins, BALF1/BCRF1/BHRF1, are expressed in EBV lytic phase and increases EBV DNA loading amount and the antibody titles (EBV viral-capsid antigen (VCA-IgA)).^53^ The plasma level of EBV DNA is considered as a distinguishing marker for NPC in subjects from healthy high-risk populations and is also a novel prognostic marker of metastatic/recurrent NPC, which complements the TNM stage classification and can be used to judge therapeutic response to radiation and chemotherapy.^53–58^ In NPC patients with low EBV DNA level, intensity modulated radiotherapy (IMRT) and concurrent chemoradiation (CCRT) are the major therapeutic strategies applied.^59^ But, in patients with a high level of EBV DNA, IMRT, CCRT, and possibly neoadjuvant chemotherapy, cisplatin or 5-Fu are needed.^59^ Recently, a prospective and population-based cohort study was conducted in southern China.^60^ In this study, serum EBV VCA-IgA antibodies and qualified EBV DNA loading from nasopharyngeal swab were used as methods to predict NPC development in seropositive high-risk individuals.^60^ In summary, early detection of NPC using markers from EBV lytic proteins or the EBV DNA is an available tool.

EBV reactivation is controlled by both host and viral factors. Cellular stresses contribute to EBV reactivation. Therefore, mechanism of EBV reactivation involves oxidative stress, inflammation (IL-6/IL-8/IL-13), angiogenesis, genomic instability and cell death.^51, 61–64^ In addition, EBV lytic proteins involve in carcinogenesis process, such as BGLF4 and BGLF5 which can promote genomic instability, BARF1 and BHRF1 which can exhibit homology to Bcl-2 and play an anti-apoptotic role. Notably, the lytic genes encode immune evasion proteins, including BNLF2a which inhibits the transporter of antigen processing, BILF1 which induces major histocompatibility complex (MHC) class I internalization and degradation.^65–67^

Targeting EBV reactivation by antioxidant compound NAC and various nature compounds, such as resveratrol, curcumin, emodin or sulforaphen, showed a good potential for inhibiting the onco-virus lytic phase.^68, 69^ We found epigallocatechin gallate inhibits EBV lytic protein expression and decreases EBV copy numbers through the AMPK pathway (unpublished data). Identifying new nature compounds to intervene the EBV reactivation and sensitizing patients to the therapy might be a good way for NPC prevention.

## Onco-functions of other EBV products

Almost all cancer hallmarks could be targeted by EBV latent genes. In EBV-associated epithelial malignancies, the latent genes, beside LMP1, there are EBNA1, EBNA2, LMP2, BARTs, miR-BARTs. The major function of EBNA1 is promoting the survival of cells upon DNA damage and beneficial for cell growth. Also EBNA1 inhibits the canonical NF-κB pathway in carcinoma cells by inhibiting IκB kinase phosphorylation.^70^ EBNA2 mimics a constitutively activated NOTCH receptors. The expression of LMP2A could be detected in more than 50% of NPC cases.^71^ LMP2 promotes cell survival, stemness, EMT, which may related to the metastatic phenotype.^72–74^ Also, LMP1 and LMP2A function cooperatively to promote carcinoma development specialy in NPC.^11, 75^

EBV can encode 44 microRNAs (miRNAs), which can drive tumor growth in vivo and could be potential serum biomarkers in NPC patients. Our group identified EBV encoded miR-BHRF1-1 potentiates viral lytic replication, which involved in host p53 down-regulation.^16^

## Precision prevention of NPC in the future

Many of the estimated cancer cases and deaths can be prevented through reducing the prevalence of risk factors.^76–78^ The 2014 AACR Cancer Progress Report states that a large proportion of cancers could be prevented by modifying factors such as cancer-causing pathogens (16%).^79^ EBV is an important and fascinating cancer causing virus. We still have many questions in need of answers. The knowledge generated regarding EBV can be used to identify risk factors, genotypes, and biomarkers that can be applied in primary prevention, early detection of cancer, and prediction of those individuals who are most likely to exhibit unfavorable cancer outcomes.^80^

1. EBV infection is listed as a Group I carcinogen category by the International Agency for Research on Cancer. NPC is an infection-related cancer strongly driven by EBV.^81^ As EBV is associated with nearly 200,000 new malignancies each year worldwide, in EBV-based primary cancer prevention, we urgently need to develop a safe and effective vaccine. Now most efforts to develop prophylactic vaccines are focused on EBV gp350. The therapeutic vaccine targeting LMP2A and EBNA1 can induce EBV-specific T cells response.^82^ None of these two types of vaccines are licensed. The major problems are lack of an animal model and are difficult to find the best immunogen and adjuvant. Prospects include prevention of infectious mononucleosis, post-transplant lymphoproliferative disease, multiple sclerosis, and treatment of EBV-related cancer.^83^
2. Smoking induces EBV lytic and smoking cessation together with EBV control might be the most important approach for the prevention of NPC. Smoking cessation programs should be advocated for the primary prevention of NPC, especially in NPC endemic areas.^54, 79^
3. Co-infection of EBV and HPV is usually found in NPC patients from endemic regions.^84^ But the co-infection of these two virus in other cancers are controversial. We need further identify the possible mechanisms of their synergistic effect during tumorigenesis and develop new strategies. Hopefully, the HPV vaccine may offer protection against a HPV-positive subtype of head-and-neck cancers in the future.^85^
4. Now the early detection and screening of the lytic proteins and EBV DNA have been applied to clinical and high-risk population. For EBV-based secondary cancer prevention, we should further confirm the effect of population-based NPC screening by EBV-related serum EBV VCA/IgA antibodies and quantitative EBV DNA loading methods, which can be used to identify the high-risk individuals and diagnose early-stage patients.^60, 86^
5. For EBV-based tertiary cancer prevention (treatment intervention), DZ1 treatment could increase the sensitivity to radiation therapy in EBV-positive NPC. Also high dose Vitamin C might reduce EBV EA IgG and VCA IgM antibody levels in NPC patients.^87^

China’s population accounts for 1/5 of the world’s population. 22% of the new cancer cases and 27% death cases are happened in China.^76^ Fortunately, China set up the ‘Health China 2030’ strategic planning. And a 3-year national plan for cancer prevention including NPC established by Chinese government is now in effect. More recently, Chinese government announced the programming “Prevention and treatment of chronic diseases in China”. The first aim of this project is to improve the cancer 5-year survival rate from 30.9 to 40.9%, and the second aim is to increase the cancer early diagnostic rate from 48 to 60% in high-risk areas before 2025. Given the importance of the contribution of EBV to the burden of the cancers especially in NPC, we should pay more attention to the EBV based cancer prevention and therapy in NPC in the future. A better understanding of the mechanism of EBV in cancer may lead to develop novel prevention and therapeutic strategies which not only for NPC, but also for EBV-associated cancers.
